# Efficacy and safety of repeated transcranial magnetic stimulation combined with escitalopram in the treatment of major depressive disorder: a meta-analysis

**DOI:** 10.3389/fpsyt.2023.1275839

**Published:** 2024-01-03

**Authors:** Zhang Liu, Sijia Yu, Youfan Hu, Ding Wang, Shuyu Wang, Zhaohui Tang, Weihong Li

**Affiliations:** Basic Medical College, Chengdu University of Traditional Chinese Medicine, Chengdu, China

**Keywords:** major depressive disorder, MDD, repeated transcranial magnetic stimulation, rTMS, escitalopram, meta-analysis

## Abstract

**Objective:**

This study was designed to systematically review the efficacy and safety of repeated transcranial magnetic stimulation (rTMS) combined with escitalopram in treating major depressive disorder (MDD).

**Methods:**

Databases including PubMed, Embase, Cochrane, Web of Science, CNKI, Wanfang, VIP Journal, and China Biomedical Literature databases were electronically searched for randomized controlled trials of rTMS combined with escitalopram intervention for MDD treatment from the inception of these databases to 27 May 2023. Two reviewers independently screened the studies, extracted the data, and assessed the quality of the included studies. R 4.2.2 was then used for a meta-analysis.

**Results:**

In total, 19 articles involving 1,032 patients were included. The results of the meta-analysis showed that Hamilton Depression Rating Scale (HAMD) scores were significantly lower in the group receiving rTMS combined with escitalopram (experimental group) than that in the control group [weighted mean difference (WMD) = −5.30, 95% confidence interval (95% CI): −6.44 to −4.17, *p* < 0.01]. The response rate of the experimental group was significantly higher than that of the control group [odds ratio (OR): 5.48; 95% CI: 3.72 to 8.07; *p* < 0.01]. No significant difference in the adverse reaction rate was observed between the two groups (OR: 1.04, 95% CI: 0.71 to 1.52, *p* = 0.82).

**Conclusion:**

Our findings suggest that rTMS combined with escitalopram can benefit patients with MDD in a safe manner, which may help in guiding clinical practice.

**Systematic review registration:**

DOI number: 10.37766/inplasy2023.11.0114, INPLASY2023110114.

## 1 Introduction

Major depression disorder (MDD) is a mental disease that presents with persistent depression and anhedonia as the core symptoms; moreover, MDD poses a heavy disease burden (1). With an accelerated pace of life and increased social pressure, the incidence of MDD has been on the rise in recent years. Furthermore, there are data to indicate that MDD affects approximately 280 million people, or 3.8% of the global population (2), and MDD has become one of the leading causes of disability worldwide (3). With a complex pathogenesis, MDD appears to be caused by a combination of genetic, environmental (such as recent negative life events), psychological (such as cognitive patterns), and biological (such as inflammation and the monoamine pathway) factors (2, 4–6).

In clinical practice, the most commonly used treatment scheme for MDD is drug therapy. Traditional antidepressants include monoamine oxidase inhibitors (MAOIs), tricyclic antidepressants (TCAs), selective 5-hydroxytryptamine (5-HT) reuptake inhibitors (SSRIs), 5-HT and norepinephrine (NE) reuptake inhibitors (SNRIs), and NE /5-HT2 and 5-HT3 receptor antagonist antidepressants (NASSAs), represented by moclobemide, imipramine, escitalopram, venlafaxine, and mirtazapine, respectively (7). International guidelines currently recommend SSRIs as the first-line treatment for most patients with MDD (8). Among these SSRIs, escitalopram is the most selective antidepressant for 5-HT transporters (9). Yan (10) found that escitalopram, which is the S-isomer of citalopram, exerts a faster effect in the treatment process, exhibits a better therapeutic effect, and leads to fewer symptoms of nausea and gastrointestinal reactions. However, due to the long-term use of antidepressants and their side effects, patients develop tolerance to existing antidepressants, thus reducing patient compliance (7). The limitations of existing treatment options for MDD have prompted the development of novel treatment options to improve patient compliance and reduce the recurrence rate of MDD.

The main target of repeated transcranial magnetic stimulation (rTMS) treatment is the dorsolateral prefrontal cortex (DLPFC). Regarding the theoretical basis herein, a previous neurofunctional imaging study demonstrated that the activity of the left prefrontal area in patients with depression is reduced, which is an important node involved in cognitive control for emotional regulation (11). Moreover, the left DLPFC can control and regulate positive emotions, while the right DLPFC can regulate and control negative emotions. It has been reported that low-frequency stimulation promotes neuronal inhibition, while higher frequencies promote neuronal excitation (12). Therefore, rTMS can be used to treat depression using high-frequency stimulation (typically 2–20 Hz) on the left DLPFC, low-frequency stimulation (1 Hz) on the right DLPFC, or bilateral alternating stimulation. Notably, the efficacy of rTMS is recognized. Berlim et al. (13) conducted a systematic review and meta-analysis of 29 randomized controlled trials (RCTs) on rTMS in patients with depression. Regarding response and remission rates, high-frequency rTMS had significant statistical and clinical differences. Furthermore, significant efficacy was demonstrated in patients with depression, and no severe adverse effects were found. Currently, the action mechanism of rTMS is being investigated, which may involve multiple aspects such as affecting the plasticity of the postsynaptic membrane by regulating various receptors in different brain regions, including 5-HT and N-methyl-D-aspartic acid (NMDA), regulating the levels of various amino acid neurotransmitters in the brain, increasing the blood flow in the frontal lobe of the brain, increasing the level of serum brain-derived neurotrophic factor (BDNF) in the hippocampus and related structures, and regulating the genes expression of neuronal excitability (14).

A previous study showed that for patients with drug-resistant depression, adding rTMS therapy after drug therapy failure can significantly improve the efficacy of antidepressants (15). The results of an RCT by Lv et al. (16) showed that in MDD treatment, the combination of rTMS and escitalopram can effectively improve the clinical efficacy of MDD and reduce the occurrence of adverse reactions. However, the study by Zhu et al. (17) showed no significant difference in the clinical efficacy and incidence of adverse reactions of rTMS combined with escitalopram for MDD compared with the control group treated with escitalopram alone. Thus, the efficacy and safety of rTMS combined with escitalopram in MDD treatment remain unclear. Hence, a meta-analysis was conducted to objectively evaluate the efficacy and safety of rTMS combined with escitalopram in treating patients with MDD, thereby providing further evidence for clinical treatment.

## 2 Methods

### 2.1 Retrieval strategy

In this study, several Chinese and English databases were searched electronically. The relevant studies were retrieved primarily from PubMed, Embase, Cochrane, Web of Science, CNKI, Wanfang, VIP, and China Biomedical Literature databases. Repetitive transcranial magnetic stimulation, rTMS, escitalopram, and depression were combined, and subject words and free words were used for the search (see Supplementary Table 1). The search time was from the inception of these databases to 27 May 2023. The search for each database was conducted independently by two reviewers.

### 2.2 Inclusion and exclusion criteria

The inclusion criteria were as follows: (1) RCTs on the intervention effect of rTMS combined with escitalopram in patients with MDD; (2) patients diagnosed with MDD combined with the International Classification of Diseases (ICD), Diagnostic and Statistical Manual of Mental Disorders (DSM), and Hamilton Depression Rating Scale (HAMD) scores; (3) the experimental group received rTMS combined with escitalopram, while the control group only received escitalopram or escitalopram combined with pseudo-stimulation; (4) the primary outcome indicators included clinical effectiveness, HAMD scores, and adverse events, while the secondary outcome indicators included Pittsburgh Sleep Quality Index (PSQI), 5-HT, norepinephrine (NE), and BDNF; and (5) Chinese and English literature.

The exclusion criteria were as follows: (1) studies with inconsistent subject and object; (2) studies with data duplication; (3) studies with full text not available and those with incomplete data; and (4) studies on subtypes of MDD (such as severe postpartum depression and severe post-stroke depression).

### 2.3 Data extraction

Endnote 20.2 was used for importing literature and screening. Two researchers screened the literature and extracted the data according to the study design and the inclusion and exclusion criteria. Differences, if any, were resolved through discussion until a consensus was reached. Otherwise, a third researcher was consulted. The basic contents of the literature were extracted, including the first author, sample size, age, main parameter indicators of rTMS (target, frequency, and intensity), diagnostic criteria, and outcomes (HAMD, clinical effectiveness, clinical effectiveness, 5-HT, BDNF, NE, PSQI, and RBANS).

### 2.4 Quality assessment

The Cochrane Handbook of Systematic Reviews (18) was followed to conduct quality reviews, including generation of random sequences, assignment concealment, blinding of participants and implementers, blinding of outcome reviews, exit and loss of follow-up, selective publication, and other risks of bias. The evaluation criteria were classified as “low risk,” “high risk,” and “unclear risk.”

### 2.5 Data analysis

Data were analyzed using R 4.2.2, and the χ^2^ test was used to assess heterogeneity. When all the studies demonstrated statistical homogeneity (*p* ≥ 0.05, *I*^2^ ≤ 50%), a fixed-effect model was used. If *p* < 0.05 and *I*^2^ > 50%, a large heterogeneity was considered present between the studies, and a random-effect model was used in this case. For the comprehensive effects analysis, weighted mean difference (WMD), odds ratio (OR), and 95% confidence interval (95% CI) were used as the effect indicators. In addition, subgroup analysis was performed according to different rTMS frequencies, intensities, stimulation sites, and ages for investigating the potential heterogeneity between the studies and the efficacy of rTMS combined with escitalopram in treating MDD. Finally, the funnel plot and Egger’s test were used to check the publication bias. A *p*-value of <0.05 was considered to be statistically significant.

## 3 Results

### 3.1 Literature search results

**Figure 1:**
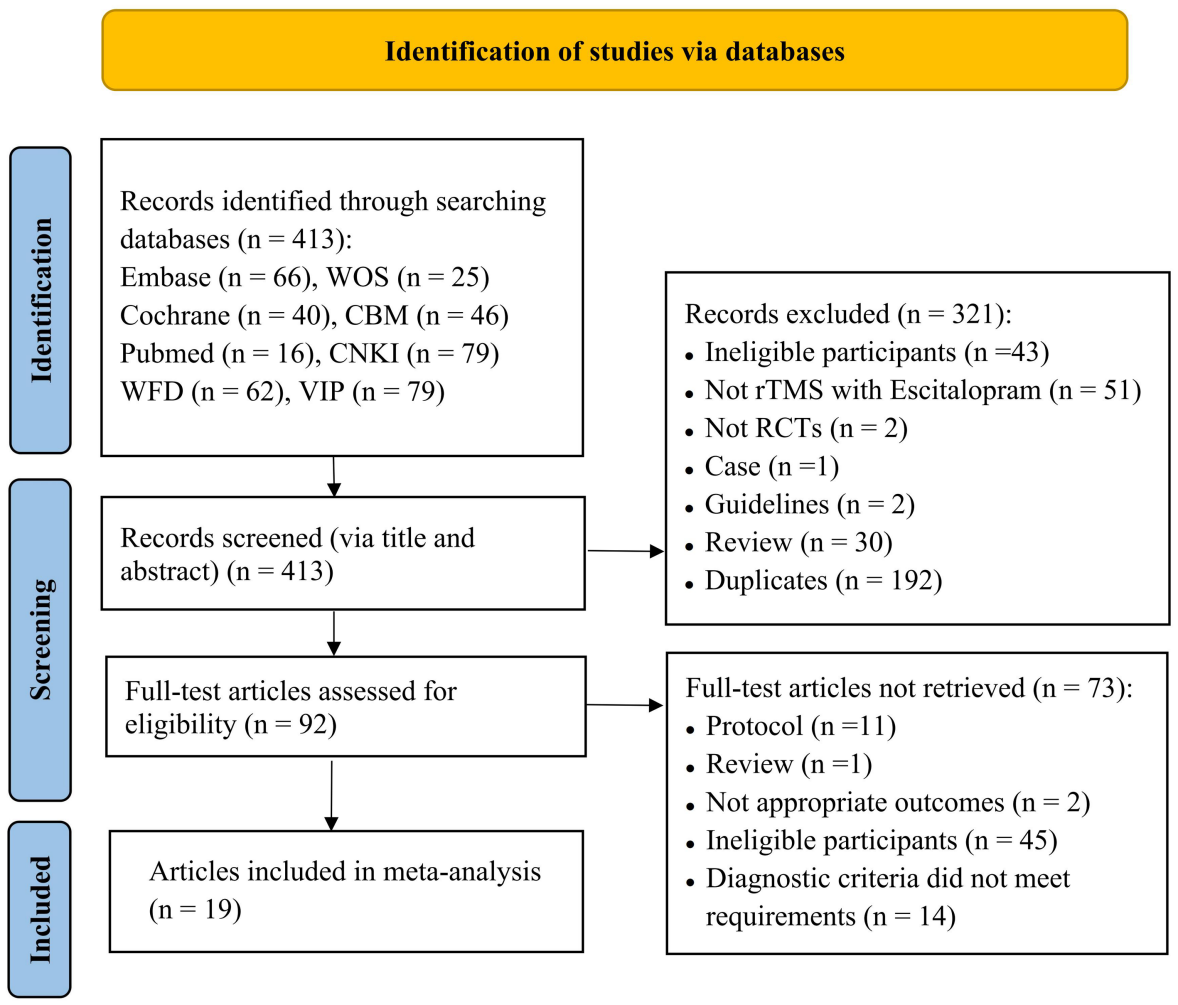
Flowchart of literature selection (CBM, Chinese Biomedical Literature Database; CNKI, Chinese National Knowledge Infrastructure; VIP, Chinese Scientific Journal Database; WFD, Wanfang Database; WOS, Web of Science).

In total, 413 articles were retrieved upon searching the aforementioned databases, of which 266 were in Chinese and 147 in English. A total of 192 duplicate articles were excluded. After reading the titles and abstracts of the articles, 229 articles were excluded. After reading the full text and screening according to the inclusion and exclusion criteria, a total of 19 articles (16, 17, 19–35) were included. Two studies in one article met the inclusion criteria, and a total of 20 studies were subjected to meta-analysis. The literature screening process is depicted in Figure 1.

**Figure 2:**
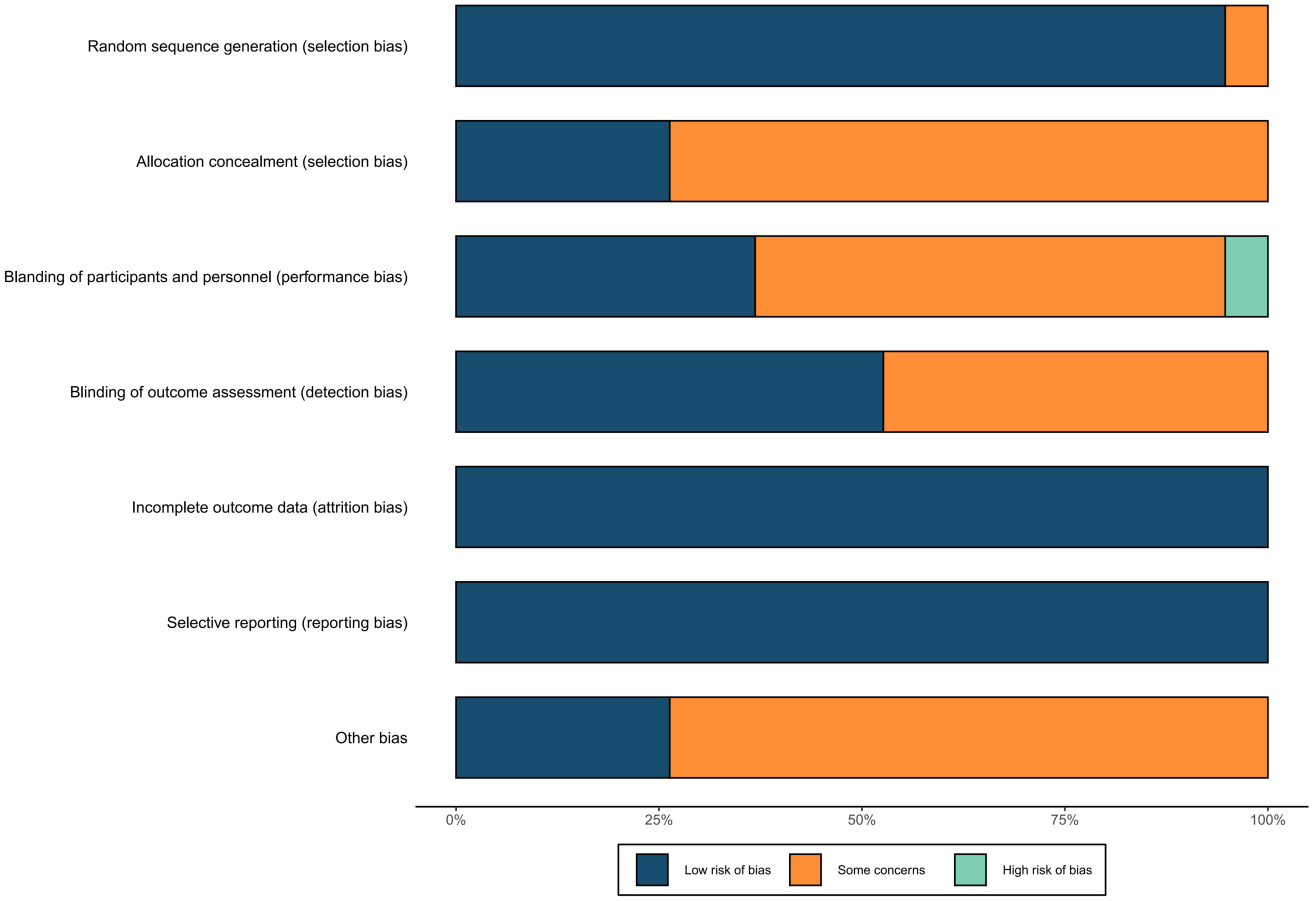
Risk of bias summary.

The basic information of the included studies is shown in Table 1. Among the 19 included articles, 3 were in English, and 16 were in Chinese. In total, 1,032 patients were enrolled, including 520 in the experimental group and 512 in the control group. All the participants were diagnosed with MDD using a combination of DSM, ICD, and HAMD scores, and all the participants received escitalopram during treatment either with rTMS, or with pseudo-stimulation (i.e., the same coil also produced a tapping sound on the scalp surface of the patient, but without a pulse), or with medication alone. The results of migration risk assessment are shown in Figure 2 and Supplementary Figure 1.

**Table 1:** Characteristics of the included studies.

Publications	Sample size (T/C)	Age (mean ± SD/range)		Target	Frequency	Intensity (% MT)	Diagnostic criteria	Outcomes
		*T*	*C*					
Bretlau et al.	22/23	53.10 ± 10.10	57.80 ± 10.00	LDLPFC	High	90%	DSM-IV	①④⑤⑥⑦⑧
Cui et al.	34/33	65.00 ± 4.70	64.00 ± 6.70	LDLPFC	High	110%	ICD-10	①②⑦
Chen	29/31	70.31 ± 4.13	71.03 ± 3.99	RDLPFC	Low	80%	ICD-10	①⑧
Chen	32/31	70.31 ± 4.13	71.03 ± 3.99	LDLPFC	High	80%	ICD-10	①⑧
Chen et al.	43/43	68.23 ± 2.28	68.15 ± 2.31	RDLPFC	Low	100%	DSM-IV	①②
Guan et al.	27/24	31.11 ± 7.65	29.00 ± 7.12	Oz point	High	120%	DSM-V	①
Fen et al.	21/21	18.14 ± 3.94	21.43 ± 6.79	LDLPFC	High	100%	DSM-IV	①④⑤⑥⑦⑧
Pang et al.	57/57	69.68 ± 7.80	70.13 ± 7.79	LDLPFC	High	90%	DSM-V	①②④⑤⑥
Gong et al.	30/30	37.00 ± 2.10	36.00 ± 2.70	LDLPFC	High	40%	ICD-10	②
Qiu et al.	24/24	34.00 ± 8.00		LDLPFC	High	80–100%	ICD-10	①②
Wang	28/26	37.25 ± 16.13	32.38 ± 17.32	LDLPFC	High	110%	ICD-10	①②⑤⑧
Wang et al.	20/18	29.90 ± 8.00	27.10 ± 5.00	Oz point	High	80%	DSM-V	①
Yang et al.	38/36	18.00–60.00		LDLPFC	High	110%	DSM-IV	②
Zhu et al.	30/30	31.70 ± 10.20	32.40 ± 12.50	RDLPFC	Low	100%	DSM-IV	①②
Zhao	30/30	72.51 ± 2.36	71.86 ± 2.46	LDLPFC	High	80%	ICD-10	①②
Zheng	19/17	26.90 ± 6.20	27.40 ± 4.80	LDLPFC	High	110%	DSM-IV	①②⑦
Zhang et al.	30/30	30.20 ± 12.50	28.70 ± 11.30	LDLPFC	High	90%	ICD-10	①②
Lv et al.	30/30	53.25 ± 5.78	54.27 ± 6.52	LDLPFC	High	100%	ICD-10	①
Wang et al.	20/18	29.90 ± 8.00	27.10 ± 5.00	Oz point	High	80%	DSM-V	①
Tang et al.	16/17	24.00 ± 4.53	24.29 ± 3.92	LDLPFC	High	90%	ICD-10	①②

C, control group; DSM, Diagnostic and Statistical Manual of Mental Disorders; ICD, The International Classification of Diseases; LDLPFC, left dorsolateral prefrontal cortex; Oz point, occipital; Outcomes (① HAMD, Hamilton Depression Rating Scale; ② Effective rate; ③ Adverse reaction rate; ④ 5-HT, 5-hydroxytryptamine; ⑤ BDNF, brain-derived neurotrophic factor; ⑥ NE, norepinephrine; ⑦ PSQI, Pittsburgh Sleep Quality Index; ⑧ RBANS, Repeatable Battery for the Assessment of Neuropsychological Status); RDLPFC, right dorsolateral prefrontal cortex; SD, standard deviation; T, treatment group.

### 3.2 Primary outcomes

#### 3.2.1 HAMD

**Figure 3:**
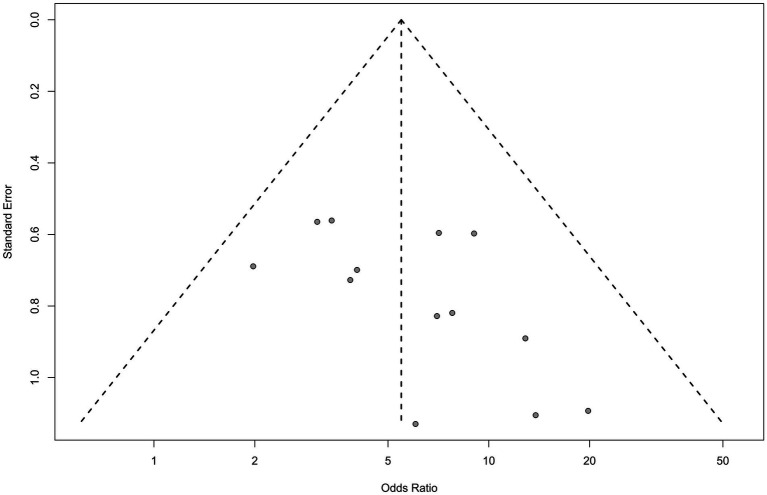
Funnel plot of HAMD.

As shown in Table 2, 18 studies (16, 17, 19–25, 27–29, 31–35) recorded the differences in HAMD scores before and after treatment. The results of the meta-analysis showed that changes in HAMD scores in the group receiving rTMS combined with escitalopram (experimental group) were significantly higher than that in the control group (WMD: −5.30, 95% CI: −6.44 to −4.17, *p* < 0.01). The heterogeneity of the studies was high [heterogeneity chi-squared (*χ*^2^) = 4.23, *p* < 0.01; *I*^2^ = 77.0%]. The funnel plot (see Figure 3) showed the presentation offset of the visual examination. The results of the Egger’s test showed no potential risk of publication bias (*t* = −1.08, *p* = 0.2971).

**Table 2:** Outcomes and subgroup analyses based on primary outcomes.

Meta-analyses variables	Number of studies	Number of patients		Pooled effect sizes	Heterogeneity	
		*T*	*C*		*P*	*I*^2^ (%)
*Dichotomous variables*				OR (95% CI)		
CE	13	409	402	5.48 (3.72 to 8.07)	0.74	0.00%
AEs	16	491	485	1.04 (0.71 to 1.52)	0.58	0.00%
*Continuous variables*				WMD (95% CI)		
HAMD	18	522	483	−5.30 (−6.44 to −4.17)	<0.01	77.00%
5-HT	2	87	87	22.76 (15.87 to 29.66)	0.02	82.00%
BDNF	3	115	113	8.82 (6.80 to 10.85)	0.89	0.00%
NE	2	87	87	3.41 (2.77 to 4.04)	0.25	23.00%
PSQI	2	53	50	−6.08 (−8.11 to −4.06)	0.64	0.00%
RBANS	3	89	88	25.62 (11.22 to 40.02)	<0.01	94.00%
*Subgroup analyses based on HAMD*				WMD (95% CI)		
*Age*						
Overall	18	522	483	−5.30 (−6.44 to −4.17)	<0.01	77.00%
<50 years	10	245	236	−5.47 (−7.80 to −3.13)	<0.01	82.00%
≥50 years	8	277	247	−5.65 (−6.46 to −4.84)	0.23	24.00%
*Intensity*						
Overall	18	522	483	−5.30 (−6.44 to −4.17)	< 0.01	77.00%
≤100%	14	414	383	−5.49 (−6.56 to −4.42)	<0.01	72.00%
110%	3	81	76	−5.64 (−8.82 to −2.46)	<0.01	79.00%
120%	1	27	24	−0.91 (−2.97 to 1.14)	/	/
*Frequency*						
Overall	18	522	483	−5.30 (−6.44 to −4.17)	<0.01	77.00%
High	14	390	366	−5.55 (−7.01 to −4.09)	<0.01	77.00%
Low	4	132	117	−4.64 (−6.42 to −2.87)	0.03	68.00%
*Target*						
Overall	18	522	483	−5.30 (−6.44 to −4.17)	< 0.01	77.00%
LDLPFC	12	343	324	−6.05 (−7.42 to −4.67)	< 0.01	67.00%
RDLPFC	4	132	117	−4.64 (−6.42 to −2.87)	0.03	68.00%
Oz point	2	47	42	−2.49 (−5.67 to 0.69)	0.04	76.00%
*Diagnostic criteria*						
Overall	18	522	483	−5.30 (−6.44 to −4.17)	<0.01	77.00%
ICD	9	253	221	−5.73 (−6.59 to −4.86)	0.23	24.00%
DSM	9	269	262	−5.21 (−7.50 to −2.92)	< 0.01	84.00%
*Subgroup analyses based on CE*				OR (95% CI)		
*Age*						
Overall	13	409	402	5.48 (3.72 to 8.07)	0.74	0.00%
< 50 years	9	245	239	5.96 (3.65 to 9.73)	0.54	0.00%
≥50 years	4	164	163	4.76 (2.53 to 8.98)	0.72	0.00%
*Intensity*						
Overall	13	409	402	5.48 (3.72 to 8.07)	0.74	0.00%
≤100%	9	290	290	4.90 (3.05 to 7.88)	0.75	0.00%
110%	4	119	112	6.86 (3.50 to 13.43)	0.39	0.00%
*Frequency*						
Overall	13	409	402	5.48 (3.72 to 8.07)	0.74	0.00%
High	10	306	300	6.23 (4.03 to 9.63)	0.74	0.00%
Low	3	103	102	3.28 (1.38 to 7.81)	0.63	0.00%
*Target*						
Overall	13	409	402	5.48 (3.72 to 8.07)	0.74	0.00%
LDLPFC	10	306	300	6.23 (4.03 to 9.63)	0.74	0.00%
RDLPFC	3	103	102	3.28 (1.38 to 7.81)	0.63	0.00%
*Diagnostic criteria*						
Overall	13	409	402	5.48 (3.72 to 8.07)	0.74	0.00%
ICD	7	192	190	7.98 (4.56 to 13.96)	0.92	0.00%
DSM	6	217	212	3.77 (2.19 to 6.51)	0.68	0.00%

AEs, adverse events; BDNF, brain-derived neurotrophic factor; C, control group; CE, clinical effectiveness; CI, confidence interval; DSM, Diagnostic and Statistical Manual of Mental Disorders; HAMD, Hamilton Depression Rating Scale; ICD, The International Classification of Diseases; LDLPFC, left dorsolateral prefrontal cortex; NE, norepinephrine; OR, odds ratio; Oz point, occipital; PSQI, Pittsburgh Sleep Quality Index; RBANS, Repeatable Battery for the Assessment of Neuropsychological Status; RDLPFC, right dorsolateral prefrontal cortex; T, treatment group; WMD, weighted mean difference; 5-HT, 5-hydroxytryptamine.

A subgroup analysis was performed according to age, stimulus intensity, stimulus frequency, target, and diagnostic criteria.

#### Age

A meta-analysis using a random effects model showed that for patients with MDD who were older than or equal to 50 years (WMD = −5.65, 95% CI: −6.46 to −4.84, *p* < 0.01) and younger than 50 years (WMD = −5.47, 95% CI: −7.80 to −3.13, *p* < 0.01), the change in HAMD scores was significantly better in the experimental group than those in the control group (see Table 2).

A meta-analysis using a random effects model showed that for patients with MDD who were older than or equal to 50 years (WMD = −5.65, 95% CI: −6.46 to −4.84, *p* < 0.01) and younger than 50 years (WMD = −5.47, 95% CI: −7.80 to −3.13, *p* < 0.01), the change in HAMD scores was significantly better in the experimental group than that in the control group (see Table 2).

#### Intensity

In the two subgroups with intensity thresholds less than or equal to 100 and 110%, the improvement in HAMD score results was evidenced by WMD = −5.49, 95% CI: −6.56 to −4.42, and p < 0.01 as well as WMD = −5.64, 95% CI: −8.82 to −2.46, and p < 0.01, respectively. The results showed that intensity of stimulation less than or equal to 100 and 110% improved the HAMD scores in patients with MDD, and this improvement was significantly better in the experimental group than in the control group (see Table 2).

#### Frequency

rTMS intervention was classified into two subgroups according to different frequencies of rTMS: rTMS frequency greater than 1 Hz was classified as the high-frequency group, and frequency less than or equal to 1 Hz was classified as the low-frequency group. Meta-analysis using the random effects model showed that in the high-frequency group (WMD = −5.55, 95% CI: −7.01 to −4.09, *p* < 0.01) and low-frequency group (WMD = −4.64, 95% CI: −6.42 to −2.87, p < 0.01), the improvement in HAMD scores were significantly better in the experimental group than that in the control group (see Table 2).

#### Target

Meta-analysis using a random effects model showed that action on left DLPFC (WMD = −6.05, 95% CI: −7.42 to −4.67, p < 0.01) and right DLPFC (WMD = −4.64, 95% CI: −6.42 to −2.87, *p* < 0.01) significantly improved HAMD scores in patients with MDD compared with the control group when treated with Oz points (WMD = −2.49, 95% CI: −5.67 to 0.69, *p* = 0.13) (see Table 2).

#### Diagnostic criteria

According to different diagnostic criteria, patients were divided into ICD and DSM subgroups. In the two subgroups, meta-analysis using a random-effects model showed that rTMS combined with escitalopram improved HAMD scores of patients with MDD compared with the control group (WMD = −5.73, 95% CI: −6.59 to −4.86, *p* < 0.01, and WMD = −5.21, 95% CI: −7.50 to −2.92, *p* < 0.01) (see Table 2).

#### 3.2.2 Clinical effectiveness

**Figure 4:**
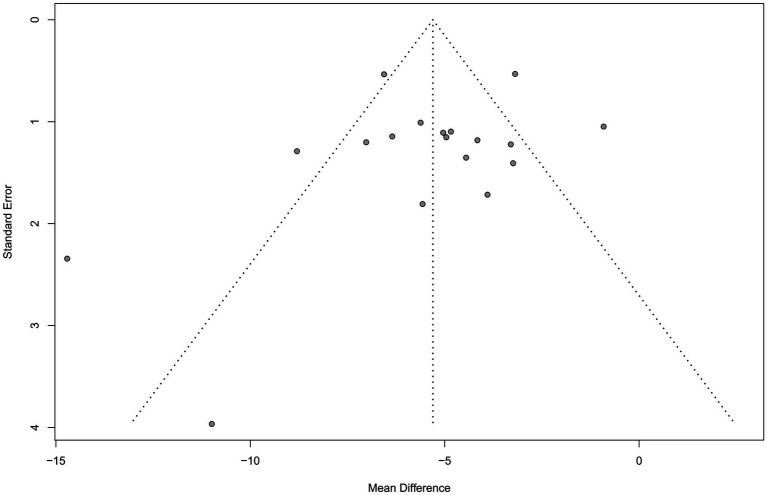
Funnel plot of CE.

The clinical effectiveness of MDD was reported in 13 studies (17, 20, 22, 25–28, 30–35) with low heterogeneity (*χ*^2^ = 0.00, *p* = 0.74; *I*^2^ = 0.00%). Meta-analysis using the fixed-effect model showed that the clinical effectiveness of the experimental group exhibited a statistically significant difference with the control group (OR: 5.48; 95% CI: 3.72 to 8.07; *p* < 0.01) (see Table 2). The funnel plot (see Figure 4) showed the presentation offset of the visual examination. The Egger’s test results showed no potential risk of publication bias (*t* = 2.19, *p* = 0.0513).

Subgroup analysis was performed according to age, stimulus intensity, stimulus frequency, target, and diagnostic criteria.

#### Age

A meta-analysis using a fixed-effect model showed that the clinical effectiveness of patients with MDD in the experimental group was significantly better than that in the control group for the two subgroups with individuals older than or equal to 50 years (OR: 4.76, 95% CI: 2.53 to 8.98, *p* < 0.01) and younger than 50 years (OR: 5.96, 95% CI:3.65 to 9.73, *p* < 0.01) (see Table 2).

#### Intensity

In the two subgroups with an intensity threshold of less than or equal to 100 and 110%, the intervention results for rTMS combined with escitalopram in patients with MDD were as follows: OR: 4.90, 95% CI: 3.05 to 7.88, and *p* < 0.01 as well as OR: 6.86, 95% CI: 3.50 to 13.43, and *p* < 0.01, respectively. The results showed that the clinical effectiveness of intensity stimulation less than or equal to 100 and 110% was significantly better than that under control treatment (see Table 2).

#### Frequency

Meta-analysis using a fixed-effect model showed that in the high-frequency group (OR: 6.23, 95% CI: 4.03 to 9.63, *p* < 0.01) and the low-frequency group (OR: 3.28, 95% CI: 1.38 to 7.81, *p* < 0.01), the clinical effectiveness of intervention in patients with MDD was significantly better than that under control treatment (see Table 2).

#### Target

Meta-analysis using a fixed-effect model showed the effect on left DLPFC (OR: 6.23, 95% CI: 4.03 to 9.63, *p* < 0.01) and right DLPFC (OR: 3.28, 95% CI: 1.38 to 7.81, *p* < 0.01). The clinical effectiveness of intervention in patients with MDD was significantly better than that under control treatment (see Table 2).

#### Diagnostic criteria

According to different diagnostic criteria, patients were divided into ICD and DSM subgroups. In the two subgroups, meta-analysis using a fixed-effects model showed that the effects of rTMS combined with escitalopram for intervention in patients with MDD were evidenced by OR: 7.98, 95%, CI: 4.56 to 13.96, and *p* < 0.01 as well as OR: 3.77, 95% CI: 2.19 to 6.51, and *p* < 0.01, respectively (See Table 2).

### 3.3 Secondary outcomes

#### 3.3.1 Serum neurotransmitter

Two studies (25, 31) reported the level of 5-HT in the experimental and control groups before and after treatment. Meta-analysis results showed that the level of 5-HT in patients with MDD treated with rTMS combined with escitalopram was significantly higher than that in the control group (WMD = 22.76, 95% CI: 15.87 to 29.66, *p* < 0.01). Three studies (25, 28, 31) reported the level of BDNF in the experimental and control groups before and after treatment, and the results of the meta-analysis showed that BDNF levels in patients with MDD treated with rTMS combined with escitalopram were significantly higher than that in the control group (WMD = 8.82, 95% CI: 6.80 to 10.85, *p* < 0.01). Two studies (25, 31) reported NE levels in the experimental and the control groups before and after treatment. Meta-analysis results showed that the NE level in patients with MDD treated with rTMS combined with escitalopram was significantly higher than that in the control group (WMD = 3.41, 95% CI: 2.77 to 4.04, *p* < 0.01) (see Table 2).

#### 3.3.2 PSQI

Two studies (20, 32) reported PSQI scores in the experimental and the control groups before and after treatment. Meta-analysis results showed that PSQI scores for patients with MDD who were treated with rTMS combined with escitalopram were significantly lower than those for patients in the control group (WMD = −6.08, 95% CI: −8.11 to −4.06, *p* < 0.01) (see Table 2).

#### 3.3.3 RBANS

Three studies (21, 28) reported the RBANS scores of the experimental and the control groups before and after treatment. Meta-analysis showed that the intervention of rTMS combined with escitalopram had significantly higher RBANS scores than those for the control group (WMD = 25.62, 95% CI: 11.22 to 40.02, *p* < 0.01) (see Table 2).

#### 3.3.4 Adverse events

In total, 16 studies (16, 17, 20–22, 24, 25, 27, 28, 30–33, 35) reported adverse events after treatment. Adverse reactions included insomnia, dry mouth, lethargy, dizziness, headache, diarrhea, fatigue, nausea, and vomiting. The adverse events were mild and disappeared without special treatment. A research study by Lv Lina et al. (16) demonstrated that during the treatment period, the adverse reaction rate of the control group was 15%. The reactions included dry mouth, nausea, dizziness, and sleepiness. The adverse reaction rate in the observation group was 7.5%, including dizziness, nausea, and drowsiness. Overall, the adverse reaction rate in the control group was significantly higher than that in the experimental group (*χ*^2^ = 5.271, *p* = 0.000).

Meta-analysis using a fixed-effects model suggested no significant differences between the experimental and the control groups in terms of the adverse reaction rate during treatment (OR: 1.04, 95% CI: 0.71 to 1.52, *p* = 0.82) (see Table 2).

## 4 Sensitivity analysis

Sensitivity analysis was performed on the HAMD scores and clinical effectiveness results of rTMS combined with escitalopram intervention for MDD. Consequently, one article was excluded, and a meta-analysis was performed on the remaining articles. The combined results of the remaining studies were still statistically significant, indicating that the results were robust and had no impact on the final results.

## 5 Discussion

This study systematically reviewed the efficacy of rTMS combined with escitalopram intervention for MDD. The results showed that the HAMD scores of the experimental group were significantly lower than those of the control group, whereas the clinical effectiveness was significantly higher than that of the control group. In addition, the results suggested no statistically significant difference in the incidence of adverse events between the experimental and the control groups. Furthermore, Lv et al. (16) showed that the addition of rTMS helped in reducing adverse events. In conclusion, our study shows that rTMS combined with escitalopram has broad prospects of application in MDD treatment, which would provide new evidence for the effectiveness and safety of rTMS combined with escitalopram in improving depressive mood in patients with MDD. Regarding the improvement of HAMD scores in patients with MDD in the experimental group, our results showed moderate heterogeneity among the included studies. To explore the possible influencing factors involved in improving HAMD scores in patients with MDD in the experimental group, a subgroup analysis was performed based on age, frequency, intensity, site of stimulation, and diagnostic criteria, which showed that these factors were indeed the source of heterogeneity in this study. Furthermore, subgroup analysis of articles involving the efficacy rate was performed according to age, frequency, intensity, stimulation site, and diagnostic criteria to accurately evaluate the efficacy of rTMS combined with escitalopram in treating MDD. The results showed that the efficacy rate of rTMS combined with escitalopram in treating MDD was significantly higher than that in the control group in different subgroups.

MDD is a common mental disease that is predicted to be one of the top three causes of the world’s disease burden by 2030 due to its relapses (36). Furthermore, MDD is the most common mental disorder. According to community surveys from 30 countries, its lifetime prevalence rate is up to 10.8% (37). The pathophysiological mechanism of depression is very complex, and the specific mechanism has not yet been fully elucidated. In recent years, the most popular view represented by the “biological mechanism hypothesis” states that the monoamine neurotransmitter system is closely related to the control of emotions and behaviors, implying that the onset, progression, and prognosis of depression are related to the abnormally low expression of 5-HT, NE, and BDNF in the brain (38). NE is a neurotransmitter primarily derived from adrenal medulla, sympathetic postganglionic neurons, and adrenergic neurons. Derived from tryptophan, 5-HT is a central neurotransmitter. BDNF belongs to a protein family of neurotrophic factors, and it plays an important role in the growth, development, differentiation, and maintenance of various types of neurons in the central nervous system (39). Therefore, the effective regulation of related neurotrophic factors and selective inhibition of neuronal reuptake of 5-HT and NE in patients with depressive disorders is particularly critical for prognosis (40). Antidepressant therapy is still the mainstream treatment for depression. Escitalopram oxalate is a commonly used selective 5-HT recovery inhibitor (SSRI) with a unique action mechanism that can inhibit 5-HT reuptake more effectively than other SSRIs as well as exert a faster treatment effect for depression. The effect of escitalopram oxalate is certain (41). However, some patients’ conditions cannot be effectively alleviated by drugs alone. Therefore, it is crucial to evaluate and develop an improved comprehensive treatment plan for improving the symptoms of patients with depression.

rTMS is a nerve stimulation and neuromodulation technology that generates electric fields in the brain based on the principle of electromagnetic induction. The magnetic field can penetrate the skull into the cerebral cortex without attenuation, change the local electrical activity of the cerebral cortex (42, 43), and then stimulate the vascular tissues and cerebral nerves, thereby accelerating the speed of cerebral microcirculation and increasing cerebral blood flow. The normal excitability of nerves is improved, and then depression symptoms are improved. Clinical practice and functional imaging have confirmed that high- and low-frequency rTMS have different effects on the physiological function of the brain: high-frequency rTMS can increase local cortical excitability, and low-frequency rTMS can decrease local cortical excitability (44). Some researchers believe that the functional activity of the left prefrontal cortex in patients with depression is reduced and the functional activity of the right is relatively hyperactive (45). Therefore, stimulating the left prefrontal cortex with high-frequency rTMS or the right prefrontal cortex with low-frequency rTMS could theoretically improve depressive symptoms. Aleman (46) found that low-frequency rTMS may activate the left brain hemisphere by inhibiting the right brain hemisphere and thereafter inhibiting the corpus callosum connection, thus leading to antidepressant effects. Previous studies have suggested that the occurrence of depression is related to the decrease of monoamine neurotransmitters 5-HT and DA and an imbalance of excitatory and inhibitory amino acids in the brain (44). Chen et al. (47, 48) found that low-frequency rTMS can regulate the levels of monoamine transmitters in different brain regions and glutamate levels in the hippocampus of depressed model rats, which may be the mechanism of low-frequency rTMS in treating depression. rTMS has a synergistic effect on antidepressant treatment and can improve the prognosis of depression. Regarding the advantages of rTMS, Fang et al. (49) and Berlim et al. (50) have reported similar observations, and they proposed that it may be attributed to the fact that rTMS can enhance the activity of 5-HT and adrenergic neurons after stimulating the corresponding brain regions, which can enhance the efficacy of antidepressants and shorten the drug onset time. Thus, the pain experienced by patients with MDD could be alleviated rapidly. Pang et al. (25) confirmed that rTMS combined with escitalopram can promote the synthesis and release of neurocytokines and monoamine neurotransmitters in patients with MDD, which is conducive to disease recovery.

It has been reported that repetitive low-frequency transcranial magnetic stimulation can help patients improve the level of neurotransmitters in the brain that affect the sleep–wake cycle and directly affect the deep brain tissue. In addition, magnetic field exposure affects the secretion and synthesis of melatonin in patients to a certain extent, thereby accelerating the recovery of the body’s normal sleep–wake cycle. Chen et al. (22) confirmed that repetitive low-frequency transcranial magnetic stimulation can significantly improve patients’ continuous attention, working memory, and cognitive processing ability. This is attributed to the fact that repetitive low-frequency transcranial magnetic stimulation can increase blood flow in the left prefrontal cortex, effectively regulate gene expression of neuronal excitability, and promote effective communication between neurons. Cognitive impairment of patients with depression results in overall damage to brain function (51), especially with damage to the frontal and temporal lobe function. The prefrontal cortex has rich connections with the temporal and parietal lobes, along with extensive fiber connections with the diencephalon, midbrain, and limbic system. Moreover, higher-order mental activities are related to the function of the prefrontal cortex. Therefore, it is currently believed that the structural changes and functional decline of the prefrontal cortex are the main reasons for cognitive impairment in patients with depression. In addition, functional imaging studies (52, 53) showed that the activities of both sides of the prefrontal cortex in patients with depression were weakened, especially on the left side. Therefore, high-frequency rTMS treatment of the left DLPFC of the brain in patients with depression can affect the excitability and blood flow activity of the local cerebral cortex and change the neurotransmitters, cytokines, and neurotrophic factors in the brain. Remodeling the cerebral cortical function to improve patients’ positive emotions can improve cognitive function.

This study aimed to explore the effect of rTMS combined with escitalopram in improving the symptoms of MDD. Based on the above discussion and the outcome indicators of our quantitative analysis, rTMS combined with escitalopram has a significant effect in improving symptoms of MDD. In addition, rTMS combined with escitalopram can promote the release of serum neurotransmitters in patients with MDD, improve sleep quality, and enhance the cognitive function of these patients. According to these studies (20, 28, 32), high-frequency rTMS with an intensity of 110% had a significant effect on MDD intervention, and patients with MDD aged 50 or older who received rTMS combined with escitalopram gained a more significant effect than patients with MDD below 50 years of age.

This study had several limitations. First, most studies did not report the blind method and allocation hiding; hence, a certain heterogeneity exists. Second, there were only few studies included in some subgroups in the subgroup analysis, and more studies need to be included in future to further verify the conclusions reached in this study. Third, regarding the effects of rTMS combined with escitalopram on promoting the release of serum neurotransmitters in patients with MDD, improving patients’ sleep quality, and improving patients’ cognitive function, few original studies were included in the current study, which may lead to false positive results. Considering the limitations of the included research studies, it is necessary to conduct multicenter, large-sample, double-blind, high-quality RCT studies to provide higher-level evidence.

Despite these limitations, to the best of our knowledge, this is the first quantitative meta-analysis of rTMS combined with escitalopram for MDD, and the sensitivity analysis proved that the final results had good stability. Hence, the results are representative, which may be an advantage of this study. In addition, this study adds to our understanding of the efficacy and safety of rTMS combined with escitalopram intervention for MDD and the improvement in serum neurotransmitters, cognitive function, and sleep performance for patients with MDD.

## Data availability statement

The original contributions presented in the study are included in the article/Supplementary material, further inquiries can be directed to the corresponding authors.

## Author contributions

ZL: Writing – original draft, Writing – review & editing. S-jY: Writing – original draft. Y-fH: Writing – original draft. DW: Writing – original draft. S-yW: Writing – original draft. Z-hT: Writing – review & editing. W-hL: Writing – review & editing.

## Supplementary material

### SUPPLEMENTARY FIGURE 1

Risk of bias graph.
