# Supplementary figures and images for: Efficacy and safety of repeated transcranial magnetic stimulation combined with escitalopram in the treatment of major depressive disorder: a meta-analysis
Source: Front Psychiatry. 2024 Jan 3;14:1275839. doi: 10.3389/fpsyt.2023.1275839 (PMC10791764; doi:10.3389/fpsyt.2023.1275839)

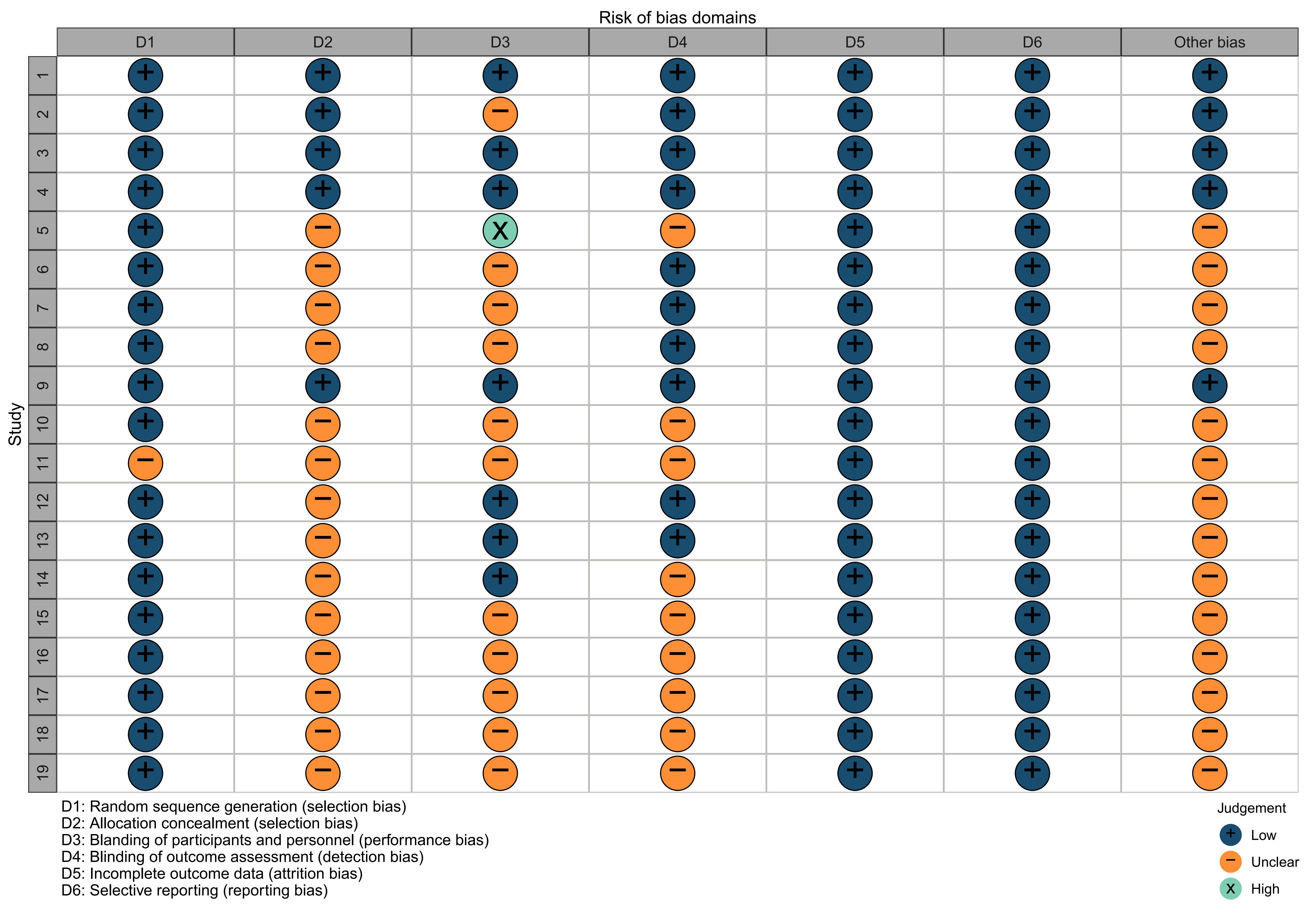

Supplement: Supplementary file 1 [file Image_1.jpeg]
