# Supplementary material for: Efficacy and safety of repeated transcranial magnetic stimulation combined with escitalopram in the treatment of major depressive disorder: a meta-analysis
Source: Front Psychiatry. 2024 Jan 3;14:1275839. doi: 10.3389/fpsyt.2023.1275839 (PMC10791764; doi:10.3389/fpsyt.2023.1275839)
Supplement: Supplementary file 2 [file Table_1.docx]

**Supplementary Table 1. Search strategies**

| **PubMed** | | |
| --- | --- | --- |
| Number | Query | Results |
| #1 | "Depression"[Mesh] | 149,290 |
| #2 | depression[Title/Abstract] OR "Depressive Symptoms"[Title/Abstract] OR "Depressive Symptom"[Title/Abstract] OR Symptom, Depressive[Title/Abstract] OR "Emotional Depression"[Title/Abstract] OR Depression, Emotional[Title/Abstract] OR "depression neurosis"[Title/Abstract] OR "depressive disorder"[Title/Abstract] OR "major depression"[Title/Abstract] OR melancholia[Title/Abstract] OR depress*[Title/Abstract] | 560,689 |
| #3 | #1 OR #2 | 584,409 |
| #4 | Escitalopram[Title/Abstract] OR "Escitalopram Oxalate"[Title/Abstract] OR Lexapro[Title/Abstract] | 3,194 |
| #5 | "transcranial magnetic stimulation"[Title/Abstract] OR TMS[Title/Abstract] OR rTMS[Title/Abstract] OR "Repetitive transcranial magnetic stimulation"[Title/Abstract] | 25,315 |
| #6 | #4 AND #5 | 23 |
| #7 | Randomized controlled trial[Publication Type] OR controlled clinical trial[Publication Type] | 683,838 |
| #8 | randomized or controlled or trial or random or placebo or groups | 7,288,919 |
| #9 | #7 OR #8 | 7,288,919 |
| #10 | #3 AND #6 AND #9 | 16 |
| **Embase** | | |
| Number | Query | Results |
| #1 | 'depression'/exp | 628,197 |
| #2 | 'symptom, depressive':ab,ti | 9 |
| #3 | 'emotional depression':ab,ti | 71 |
| #4 | 'depression, emotional':ab,ti | 316 |
| #5 | 'melancholia':ab,ti | 1,784 |
| #6 | 'depressive symptoms':ab,ti | 82,642 |
| #7 | 'depressive symptom':ab,ti | 4.535 |
| #8 | 'symptom, depressive':ab,ti | 9 |
| #9 | 'depression neurosis':ab,ti | 25 |
| #10 | 'major depression':ab,ti | 34,468 |
| #11 | #1 OR #2 OR #3 OR #4 OR #5 OR #6 OR #7 OR #8 OR #9 OR #10 | 638,298 |
| #12 | 'escitalopram'/exp | 15,382 |
| #13 | 'escitalopram oxalate'/exp OR 'escitalopram oxalate' OR lexapro:ab,ti | 15,435 |
| #14 | #12 OR #13 | 15,435 |
| #15 | 'transcranial magnetic stimulation'/exp OR 'transcranial magnetic stimulation' | 33,659 |
| #16 | 'tms'/exp OR tms OR rtms OR 'repetitive transcranial magnetic stimulation':ab,ti | 30,630 |
| #17 | #15 OR #16 | 42,873 |
| #18 | 'randomized controlled trial'/exp OR 'randomized controlled trial' OR 'controlled clinical trial':it | 1032,446 |
| #19 | #11 AND #14 AND #17 AND #18 | 66 |
